# The Impact of Obesity on Operative Outcomes and Long-Term Oncological Outcomes Following Rectal Cancer Surgery: A Retrospective Single-Center Study

**DOI:** 10.3390/jcm15072693

**Published:** 2026-04-02

**Authors:** Dan Assaf, Nadav Elbaz, Yasmin Anderson, Ofir Gruper, Eyal Mor, Michael Goldenshluger, Lior Segev

**Affiliations:** 1 Division of Surgery, The Chaim Sheba Medical Center, Tel-Hashomer, Ramat Gan 52621, Israellior.segev@sheba.health.gov.il (L.S.); 2 Gray Faculty of Medicine, Tel-Aviv University, Tel-Aviv 69978, Israel

**Keywords:** rectal cancer, obesity, outcomes

## Abstract

**Background:** Our aim was to evaluate the impact of obesity on operative and long-term oncological outcomes following rectal cancer surgery. **Methods:** Single-center retrospective review of all patients that underwent rectal resection for primary rectal adenocarcinoma between 2010 and2020. Divided into two groups based on preoperative body mass index (BMI): obese—BMI ≥ 30 kg/m^2^, non-obese—BMI < 30 kg/m^2^. **Results:** Total of 513 patients included 100 in the obese group (mean BMI 33.5 kg/m^2^, range 30–45.6) and 413 in the non-obese group (mean BMI 24.9 kg/m^2^, range 15.6–29.9, *p* < 0.001). The groups exhibited a similar age distribution (63.94 and 63.23, *p* = 0.59) or gender distribution (*p* = 0.83) between the groups. The obese group had higher conversion rates to open approach (22% versus 11.6%, *p* = 0.007), and longer surgeries (median 300 min, versus 260 min, *p* = 0.003), but similar overall postoperative complications (73% versus 64.4%, *p* = 0.1), 30-day readmission (19% versus 17.4%, *p* = 0.71), harvested lymph nodes (16 versus 14, *p* = 0.38), incidence of positive resection margins, 5-year overall survival (94.2% versus 88.7%, *p* = 0.97), and 5-year disease-free survival (70.4% versus 72%, *p* = 0.59). **Conclusions:** Obesity did not affect immediate overall surgical outcomes, surgical pathology quality indexes or long-term oncological outcomes.

## 1. Introduction

Radical rectal resection remains the primary curative modality for rectal cancer, despite the multidisciplinary approach that characterizes its modern management [1,2]. Rectal cancer surgery poses unique technical challenges due to the confined bony pelvis, the need for precise dissection, and the proximity to critical anatomical structures, all of which contribute to relatively high surgical morbidity [3]. Obesity is thought to further exacerbate these technical difficulties. Increased visceral adiposity may further restrict the already limited pelvic workspace, while a bulky mesentery can impair bowel mobility, obscure dissection planes, and limit the ability to achieve a tension-free anastomosis. Additionally, a thickened abdominal wall may complicate stoma creation [4]. Obese patients also present perioperative challenges, including difficulties with positioning, prolonged steep Trendelenburg during minimally invasive surgery, increased risk of positioning-related nerve injuries, challenging abdominal access, and issues related to airway management and vascular access [5]. In parallel with the global rise in obesity [6] and its suggested association with colorectal carcinogenesis [7,8,9,10], surgeons are increasingly encountering obese patients with rectal cancer. Despite these recognized challenges, the clinical evidence regarding how obesity dictates the operative and oncological trajectory of rectal cancer treatment remains controversial. Several studies have reported higher conversion rates to open surgery and worse short-term outcomes in obese patients [11,12], whereas others have found no significant differences in postoperative morbidity or mortality after adjustment for confounders [13,14]. Furthermore, the effect of obesity on the oncological quality metrics, such as lymph node yield, circumferential resection margin status, and completeness of total mesorectal excision (TME), remains uncertain. Importantly, the literature on long-term oncological outcomes is conflicting. Some studies suggest that obesity is associated with diminished disease-free survival and a greater risk of regional recurrence. For example, Choi et al. reported significantly lower 3-year local control in obese patients, identifying obesity as an independent predictor of local recurrence [15,16]. In contrast, other studies have demonstrated improved overall survival within cohorts classified as overweight or obese compared to those with normal or low BMI, a phenomenon sometimes attributed to differences in patient selection, tumor biology, or treatment strategies such as neoadjuvant therapy use and BMI stratification [17].

Given these inconsistencies, the present study aimed to evaluate how obesity (BMI ≥ 30 kg/m^2^) influences perioperative outcomes, pathological quality, and long-term oncological outcomes for individuals undergoing rectal cancer resection. The aim of our study was to evaluate the impact of obesity on operative complexity, including both acute and longitudinal clinical endpoints.

## 2. Materials and Methods

### 2.1. Study Design/Population

This was a single-center study conducted at a tertiary referral medical center in Israel, serving a heterogeneous population representative of the broader Middle Eastern region. The patient population includes individuals from diverse ethnic, cultural, and socioeconomic backgrounds, enhancing the generalizability of the findings. After the approval of the institutional review board (approval number 9618-22-SMC), consecutive patients undergoing radical rectal resection from 2010 to 2020 were identified via electronic medical record queries. Relevant surgical procedures, including anterior resection, proctectomy with colo-anal anastomosis, and abdomino-perineal resection (APR), were extracted using standardized coding. The final study population comprised only those with clinically staged I–III primary non-metastatic rectal adenocarcinoma. We excluded the following:

- Non-elective;
- Emergency cases;
- Palliative procedures;
- Patients lacking BMI data (13 patients).

The patient selection process is summarized in Supplementary Figure S1 (CONSORT flow diagram), resulting in a final study cohort of 513 patients, including 100 obese (BMI ≥ 30 kg/m^2^) and 413 non-obese (BMI < 30 kg/m^2^) individuals.

BMI was measured on the day admitted to surgery and was defined as weight divided by height squared (kg/m^2^). The World Health Organization (WHO) defines normal-range BMI between 18.5 and 24.9. BMI ≥ 25 is defined as overweight, BMI levels between 25 and 29.9 are considered pre-obesity, BMI levels between 30 and 34.9 are defined as obesity class 1, BMI levels between 35 and 39.9 are obesity class 2 (severe obesity), and BMI ≥ 40 is obesity class 3 (morbid obesity) [18]. We divided our cohort into two groups according to the standard WHO definition of obesity:

Obese group—BMI ≥ 30 kg/m^2^;
Non-obese group—BMI < 30 kg/m^2^.

A predefined subgroup analysis was performed to further evaluate the impact of extreme BMI categories. The cohort was stratified into severely obese patients (BMI > 35 kg/m^2^) (n = 25) and normal-weight patients (BMI < 25 kg/m^2^) (n = 209). Comparative analyses between these groups were conducted to assess differences in operative complexity, perioperative outcomes, pathological parameters, and long-term oncological outcomes.

### 2.2. Study Variables and Outcome Measures

A power analysis for a two-tailed hypothesis indicated that a total sample size of 385 patients, with a 4:1 allocation ratio (77 vs. 308), would be required to detect an effect size corresponding to a 40 min difference in operative duration (SD 112 min), with 80% power at a significance level (α) of 0.05.

Patient demographics and clinical characteristics included age, sex, BMI, smoking history, prior surgical interventions, comorbidities, and American Society of Anesthesiologists (ASA) physical status scores. Preoperative disease assessment encompassed clinical presentation, diagnostic work-up, and baseline laboratory values. Tumors were categorized by their distance from the anal verge: (lower rectum ≤ 5 cm, mid rectum 5–10 cm, upper rectum > 10 cm). Clinical staging and the administration of neoadjuvant therapy were also documented. Locally advanced disease (T3 or node-positive) served as the primary indication for neoadjuvant therapy. Treatment strategies were determined via multidisciplinary team (MDT) consensus and included the following: Short-course radiotherapy (SCRT): 25 Gy delivered in 5 fractions over one week; Long-course chemoradiotherapy (LCCRT): Typically 50.4 Gy in 25 fractions combined with 5-FU-based chemotherapy; and Total Neoadjuvant Therapy (TNT): LCCRT integrated with systemic chemotherapy (FOLFOX, FOLFIRI, or XELOX). TNT was introduced toward this study’s conclusion and was generally reserved for aggressive disease features, such as T4 stage, N2 status, or mesorectal fascia involvement. Immunotherapy was not utilized during the study period. The surgical cohort underwent abdomino-perineal resection (APR), low anterior resection (LAR) with total mesorectal excision (TME), or anterior resection (AR) with tumor-specific TME. Operative data included procedure duration, stoma creation, concomitant procedures, anastomosis technique, and conversion to open surgery. While the use of a diverting ileostomy was at the lead surgeon’s discretion, it was typically indicated by frailty, prior neoadjuvant therapy, or high-risk anastomoses (e.g., coloanal or distal resections). Short-term outcomes included hospital length of stay (LOS), 30-day readmission, and postoperative complications. Complication severity was classified using the Clavien-Dindo system, with “major complications” defined as grade >2 [19]. Histopathological evaluation focused on tumor dimensions, degree of differentiation, pathological stage, lymph node yield, and margin status. Follow-up data included adjuvant therapy details, recurrence (local or distant), and mortality. Disease-free survival (DFS) was defined as the interval between surgery and the first recorded recurrence or the last follow-up. Overall survival (OS) was calculated from the date of surgery until death or the most recent clinical contact.

Patients with missing BMI data were excluded from the analysis. Given the small number of such cases, a complete-case analysis approach was considered appropriate.

### 2.3. Statistical Analysis

Data were gathered into a REDCap database. Data analysis was performed using SPSS version 29 software (IBM Corp, Armonk, NY, USA). All statistical analyses were conducted with a significant level of α = 0.05. Continuous variables (age, BMI, preoperative albumin level, preoperative hemoglobin level, distance from the anal verge, pathological distance from the resection margin, and pathological tumor size) were assessed for distribution using skewness and kurtosis values, with absolute values below 2.0 considered approximately normal. Normally distributed continuous variables are presented as means and standard deviations and were compared between groups using Student’s *t*-test. All other variables were treated as categorical and are presented as frequencies and percentages. Categorical variables were compared using the χ^2^ test or Fisher’s exact test, as appropriate.

Univariate analysis for association with severe complications was performed using binary logistic regression, and multivariate analysis for association with severe complications was performed using a binary logistic regression model adjusting for clinically relevant covariates (age, gender, BMI, and tumor location). Variables for the multivariable model were selected based on clinical relevance and published evidence and were restricted to preoperative confounders.

Survival analysis was conducted using Kaplan–Meier curve and the log rank test for significance. Multivariate survival analysis was performed using the cox regression test adjusting for clinically relevant covariates (age, gender, BMI, tumor stage, and tumor location). Variables for the multivariable model were selected based on clinical relevance and published evidence and were restricted to preoperative confounders.

## 3. Results

### 3.1. Patients Characteristics and Demographics

The cohort included a total of 513 patients, of whom 100 patients were in the obese group (mean BMI 33.5 kg/m^2^, range 30–45.6) and 413 patients in the non-obese group (mean BMI 24.9 kg/m^2^, range 15.6–29.9, *p* < 0.001). No differences in mean age (63.94 and 63.23 in the obese and non-obese groups, respectively, *p* = 0.59) or gender distribution (*p* = 0.83) were noted between the groups. The obese patients had a higher comorbidity burden than the non-obese patients as reflected by the higher rates of ASA score 4 (6% vs. 1.9% respectively, *p* = 0.03) and a higher overall prevalence of comorbidities (90% vs. 76% respectively, *p* = 0.002). Specifically, there was a higher prevalence of metabolic syndrome- related morbidities, including hypertension, diabetes and dyslipidemia (Table 1). On the other hand, fewer patients in the obese group were actively smoking (10% vs. 20.6% respectively, *p* = 0.01).

### 3.2. Disease Presentation

Disease presentation was comparable between the groups, along with tumor location in the rectum, pre-treatment clinical stage distribution, neoadjuvant therapy protocols, and preoperative laboratory values including tumor markers levels (Table 2).

### 3.3. Surgical Procedure

The distribution of open, laparoscopic or robotic approaches was similar between the groups; however, the conversion rates from minimally invasive to open surgery were significantly higher in the obese group (22% vs. 11.6% respectively, *p* = 0.007). As a result, there were higher rates of midline laparotomy incisions in the obese group (53% vs. 37%, respectively, 0.003). Apart from the approach, the surgical procedures and the extent of resection were similar between the groups, including similar rates of abdomino-perineal resections (9% and 5.6%, respectively, *p* = 0.2), similar prevalence of additional procedures during surgery (19% and 15%, *p* = 0.33), and similar rates of stoma formation (65% and 61.3%, *p* = 0.49). In addition, there were no significant differences in intraoperative complication rates (15% and 15.7%, respectively, *p* = 0.86); however, the median surgery duration was significantly longer among the obese patients (300 min, range 100–960, compared with 260 min, range 86–600 in the non-obese, *p* = 0.003) (Table 3).

### 3.4. Postoperative Surgical Outcomes

No marked disparities were observed across the study groups regarding the cumulative postoperative complication rate (73% and 64.4%, respectively, *p* = 0.1). However, obese patients had higher rates of surgical site infection (23%) compared with the non-obese group (13.3%, *p* = 0.02). The length of hospital stay, severe complication rate, readmission rate, and 30-day mortality were comparable across the study cohorts (Table 3). Multivariate regression analysis identified an open surgical approach and preoperative albumin levels to be significantly associated with severe postoperative complications. Specifically, an upfront open approach was associated with a 2.57-fold increased risk of severe complications compared with a minimally invasive approach. BMI levels were not associated with severe complications, which are summarized in Table 4.

### 3.5. Histopathological Results

The pathological results were similar between the groups, including the rates of pathological complete response (11% in the obese and 10.4% in the non-obese, *p* = 0.86), median number of harvested lymph nodes (16 and 14 respectively, *p* = 0.38), and the incidence of positive surgical margins (Table 5).

### 3.6. Long-Term Oncological Outcomes

**Figure 1:**
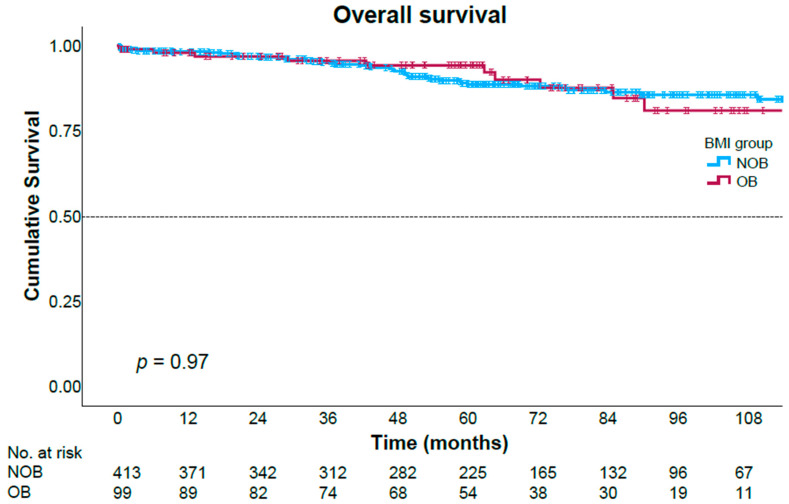
Overall survival Kaplan–Meier curves stratified by the study groups. OB—Obese group, NOB—Non-obese group.

**Figure 2:**
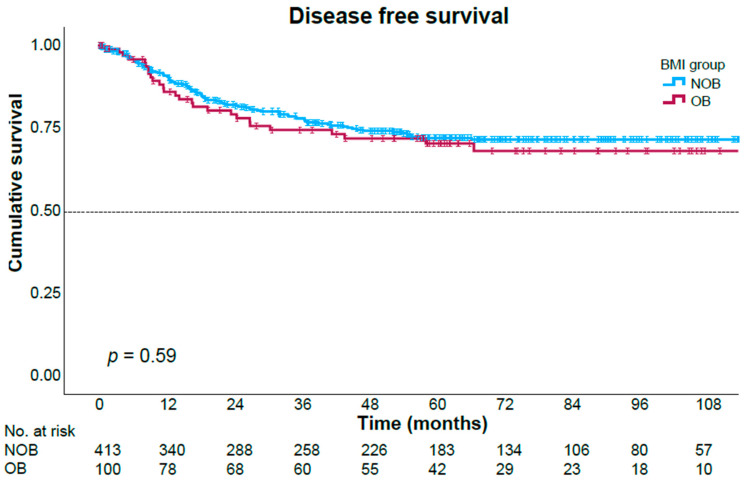
Disease-free survival Kaplan–Meier curves stratified by the study groups. OB—Obese group, NOB—Non-obese group.

After a median follow-up time of 62.6 months (range 0.6–182), we found no significant differences in overall survival nor in disease-free survival patterns between the two study groups (Figure 1 and Figure 2). The 5-year overall survival rates were 94.2% in the obese group and 88.7% in the non-obese group (*p* = 0.97), and 5-year disease-free survival rates were 70.4% and 72%, respectively (*p* = 0.59). Cox regression analysis identified pathologic tumor stage to be significantly associated with disease recurrence in both univariate and multivariate analyses (Table 6). Elevated preoperative CEA levels, distal tumor location in the rectum, and preoperative albumin levels were associated with disease recurrence only in univariate analysis. BMI levels were not associated with disease recurrence.

### 3.7. Subgroup Analysis

To validate our results, we performed a subgroup analysis comparing severely obese patients (BMI > 35, n = 25) and normal-weight patients (BMI < 25, n = 209). These groups also showed similar rates of severe postoperative complications (*p* = 0.55), overall survival (*p* = 0.76), and disease-free survival (*p* = 0.45).

## 4. Discussion

According to our data, performing rectal cancer surgery on individuals with obesity entails substantially greater operative challenges compared to non-obese cohorts, as reflected by longer operative times and higher conversion rates. Given that baseline tumor characteristics, neoadjuvant therapy utilization, and procedure types were well-balanced between groups, this increased complexity is most likely attributable to obesity itself. These findings are consistent with prior reports, including the meta-analysis by Qiu et al. [20] and large database studies [21], which have consistently shown higher conversion rates and prolonged operative duration with increasing BMI. Collectively, these data reinforce the widely held perception among surgeons that obesity significantly increases the technical difficulty of proctectomy. These findings support surgeons’ claims that obesity increases the technical difficulty of proctectomy.

Several mechanisms may explain this increased complexity, including limited pelvic workspace due to visceral adiposity, impaired visualization, and more challenging dissection planes. However, despite these intraoperative challenges, our findings, and those of others, suggest that surgical expertise and adherence to standardized total mesorectal excision (TME) principles can mitigate these difficulties. In high-volume centers, structured surgical approaches, careful preoperative planning, and appropriate patient selection likely play a key role in maintaining surgical quality, even in technically demanding obese patients.

Therefore, surgeons, especially those at the beginning of their learning curve, should be well aware of the technical implication of obesity and properly prepare before performing proctectomy in obese patients. This includes preparing the operating room setup, table, and equipment suitable for a prolonged procedure in obese patients; planning the procedure stepwise in advance; defining clear parameters for conversion; allocating additional operating room time; and ensuring the availability of an experienced rectal surgeon for assistance if needed.

Interestingly, the increased technical difficulty observed in obese patients did not translate into higher overall postoperative morbidity in our cohort. Surgical site infection (SSI) was the only complication significantly associated with obesity, a finding consistently reported in previous studies [4,20,21,22] which have shown higher rates of postoperative complications, primarily driven by SSI. This association may be partially attributed to a greater frequency of transitioning to open procedures, increased wound depth, and impaired tissue perfusion in obese patients. From a clinical perspective, these findings highlight the importance of implementing targeted preventive strategies, including meticulous wound care, optimized perioperative antibiotic protocols, and enhanced postoperative surveillance in this population.

The link between excessive body weight and other postoperative complications remains less clear. While certain reports have highlighted increased frequencies of respiratory adverse events and anastomotic dehiscence in patients with an elevated BMI [20,23], others have not confirmed these associations [21,24,25]. This inconsistency likely reflects heterogeneity in study populations, surgical techniques, and perioperative management. Emerging evidence also suggests that obesity may predispose patients to postoperative sepsis through mechanisms such as chronic low-grade inflammation, altered immune response, and impaired tissue oxygenation.

In recent years, attention has also shifted toward the identification of biomarkers that may predict postoperative complications. Novel markers such as butyrylcholinesterase (BuChE) have been proposed as potential indicators of systemic inflammatory response and postoperative risk stratification in colorectal surgery [26]. The incorporation of such biomarkers into clinical practice may improve the early detection of complications and guide personalized perioperative care strategies.

Importantly, despite increased operative complexity, our study demonstrates that oncological quality is preserved in obese patients, as evidenced by comparable lymph node yield and resection margin status. This finding is critical, as these parameters are key determinants of long-term oncological outcomes. These findings are consistent with previous studies [13,20,21,23]. One potential explanation is the “protective cushion” effect of mesorectal fat, as previously suggested by Emile et al. [27], whereby increased adipose tissue may provide a mechanical barrier to circumferential margin involvement. Additionally, the use of standardized TME techniques and surgeon expertise likely play a central role in ensuring adequate oncological resection regardless of BMI.

Consistent with most of the existing literature [12,23,27,28,29,30], our analysis revealed that overall survival (OS) and disease-free survival (DFS) were comparable between the obese and non-obese cohorts, with no statistically significant disparities observed. These findings support the notion that obesity, while increasing technical difficulty, does not compromise long-term oncological outcomes. This may be partly explained by the preservation of key oncological quality indicators, including lymph node harvest and margin negativity.

Beyond surgical outcomes, advances in technology are also shaping the future of colorectal cancer care. Notably, deep learning architecture has demonstrated considerable efficacy in the detection and categorization of colorectal malignancies from histopathological images, with the potential to enhance diagnostic accuracy and efficiency. While still evolving, these technologies may complement surgical and pathological assessment in the future.

This study is subject to several limitations, including its retrospective nature as a single-center design and the potential for selection. Although missing data were minimal and groups were generally well balanced, residual confounding cannot be excluded. Additionally, despite comprehensive data collection, details regarding mesorectal excision quality and intraoperative metrics, specifically blood loss, were unavailable for analysis, representing a constraint of this study.

Future research should focus on prospective, multicenter studies incorporating more precise measures of adiposity, such as visceral fat quantification using computed tomography, rather than relying solely on BMI. Furthermore, investigating the role of metabolic syndrome components and their interaction with surgical outcomes may provide a more nuanced understanding of risk stratification in this population.

## 5. Conclusions

In conclusion, obese patients undergoing rectal cancer surgery present with a higher comorbidity burden, and their procedures are more complex and prolonged compared with non-obese patients. Despite this, obese patients demonstrate comparable overall short-term surgical outcomes, similar pathological oncological quality, and equivalent long-term oncological outcomes. Surgical site infection was the only postoperative complication, demonstrating a significantly higher incidence within the obese cohort. Importantly, obesity was not associated with major postoperative morbidity or cancer recurrence.

## Figures and Tables

**Table 1:** Patient characteristics.

Variable	All Cohort (n = 513)	Obese Group (n = 100)	Non-Obese Group (n = 413)	*p* Value
Age in years, mean (range)	63.52 (31–88)	63.94 (33–87)	63.23 (31–88)	0.59
Gender				0.83
Male, n (%)	287 (55.9%)	55 (55%)	232 (56.2%)	
Female, n (%)	226 (44.1%)	45 (45%)	181 (43.8%)	
BMI, mean kg/m^2^ (range)	26.62 (15.6)	33.51 (30–45.6)	24.96 (15.6–29.9)	**<0.001**
Past smoking, n (%)	101 (19.7%)	21 (21%)	80 (19.4%)	0.71
Current smoking, n (%)	95 (18.5%)	10 (10%)	85 (20.6%)	**0.01**
ASA score				
Median (range)	3 (1–5)	3 (1–4)	3 (1–5)	0.14
1, n (%)	19 (3.7%)	2 (2%)	17 (4.1%)	
2, n (%)	153 (29.8%)	26 (26%)	127 (30.8%)	
3, n (%)	255 (49.7%)	48 (48%)	207 (50.1%)	
4, n (%)	14 (2.7%)	6 (6%)	8 (1.9%)	
5, n (%)	1 (0.2%)	0 (0%)	1 (0.2%)	
Missing, n (%)	71 (13.8%)	18 (18%)	53 (12.8%)	
Comorbidities prevalence				
Any, n (%)	404 (78.8%)	90 (90%)	314 (76%)	**0.002**
CVA/TIA, n (%)	18 (3.5%)	0	18 (4.4%)	**0.03**
Asthma/COPD, n (%)	48 (9.4%)	15 (15%)	33 (8%)	**0.03**
IHD/CHF, n (%)	71 (13.8%)	13 (13%)	58 (14%)	0.79
Arrythmia, n (%)	28 (5.5%)	6 (6%)	22 (5.3%)	0.79
DM, n (%)	113 (22%)	37 (37%)	76 (18.4%)	**<0.001**
CKD, n (%)	17 (3.3%)	3 (3%)	14 (3.4%)	0.99
HTN, n (%)	226 (44.1%)	61 (61%)	165 (40%)	**<0.001**
Dyslipidemia, n (%)	177 (34.5%)	50 (50%)	127 (30.8%)	**<0.001**
Family hx CRC				
1st degree, n (%)	75 (14.6%)	15 (15%)	63 (15.3%)	0.94
Non 1st degree, n (%)	42 (8.2%)	8 (8%)	34 (8.2%)	0.94
Surgical hx, n (%)	308 (60%)	58 (58%)	250 (60.5%)	0.64
Bowel surgery hx, n (%)	55 (10.7%)	19 (19%)	36 (8.7%)	**0.003**

Bold marks significance < 0.05. BMI—body mass index, ASA—American Society of Anesthesiologists, CVA—cerebrovascular accident, TIA—transient ischemic attack, COPD—chronic obstructive pulmonary disease, IHD—ischemic heart disease, CHF—congestive heart failure, DM—diabetes mellitus, CKD—chronic kidney disease, HTN—hypertension, CRC—colorectal cancer.

**Table 2:** Disease presentation.

Variable	All Cohort (n = 513)	Obese Group (n = 100)	Non-Obese Group (n = 413)	*p* Value
Symptoms present, n (%)	412 (80.3%)	74 (74%)	338 (81.8%)	0.08
Abdominal pain, n (%)	90 (17.5%)	12 (12%)	78 (18.9%)	0.10
Anemia, n (%)	45 (8.8)	9 (9%)	36 (8.7%)	0.93
Weight loss, n (%)	104 (20.3%)	12 (12%)	92 (22.3%)	**0.022**
BM change, n (%)	177 (42.9%)	39 (39%)	177 (42.9%)	0.48
LGIB, n (%)	298 (58.1%)	54 (54%)	244 (59.1%)	0.36
Tumor location				0.87
Lower rectum, n (%)	59 (11.5%)	10 (10%)	49 (11.9%)	
Mid rectum, n (%)	168 (32.7%)	33 (33%)	135 (32.7%)	
Upper rectum, n (%)	286 (55.8%)	57 (57%)	229 (55.4%)	
Distance from AV, mean (range)	9.99 (0–15)	10.07 (2–15)	9.93 (0–15)	0.75
Clinical stage				0.44
Stage 1, n (%)	117 (22.8%)	26 (26%)	91 (22%)	
Stage 2, n (%)	102 (19.9%)	19 (19%)	83 (20.1%)	
Stage 3, n (%)	253 (49.3%)	44 (44%)	209 (50.6%)	
missing, n (%)	41 (8%)	11 (11%)	30 (7.3%)	
T stage				0.54
T1, n (%)	46 (9%)	9 (9%)	37 (9%)	
T2, n (%)	98 (19.1%)	20 (20%)	78 (18.9%)	
T3, n (%)	292 (56.9%)	56 (56%)	236 (57.1%)	
T4, n (%)	36 (7%)	4 (4%)	32 (7.7%)	
missing, n (%)	41 (8%)	11 (11%)	30 (7.3%)	
N stage				0.41
N0, n (%)	229 (44.6%)	47 (47%)	182 (44.1%)	
N1, n (%)	182 (35.5%)	33 (33%)	149 (36.1%)	
N2, n (%)	71 (13.8%)	11 (11%)	60 (14.5%)	
missing, n (%)	31 (6%)	9 (9%)	22 (5.3%)	
Rectal wall tumor location				0.22
Anterior, n (%)	106 (20.7%)	23 (23%)	83 (20.1%)	
Posterior, n (%)	102 (19.9%)	23 (23%)	79 (19.1%)	
Circumferential, n (%)	77 (15%)	10 (10%)	67 (16.2%)	
Neoadjuvant therapy				
Long course chemoradiation, n (%)	215 (41.9%)	36 (36%)	169 (40.9%)	0.36
Short course radiation, n (%)	33 (6.4%)	6 (6%)	27 (6.5%)	0.84
TNT, n (%)	19 (3.7%)	4 (4%)	15 (3.6%)	0.77
Adjuvant chemotherapy, n (%)	246 (47.9%)	44 (44%)	202 (48.9)	0.38
Preop CEA, median (range)	2 (0–175)	2.1 (0–175)	1.9 (0–140.9)	0.42
Preop CA 19-9, median (range)	9.3 (0–248)	8.4 (0–92.1)	9.7 (0–248)	0.17
Preop Albumin, mean g/dL (range)	4.06 (2.4–5.1)	4.11 (3.4–4.7)	4.05 (2.4–5.1)	0.19
Preop Hgb, mean g/dL (range)	12.68 (7.76–17.2)	12.96 (9.1–16.6)	12.6 (7.76–17.2)	0.051
Preop Creatinine, median (range)	0.84 (0.2–2.49)	0.87 (0.43–1.44)	0.83 (0.2–2.49)	0.1

Bold marks significance < 0.05. CEA—carcinoembryonic antigen, BM—bowel movement, LGIB—lower gastrointestinal bleeding, AV—anal verge, TNT—total neoadjuvant therapy, Hgb—hemoglobin.

**Table 3:** Operative and perioperative details.

Variable	All Cohort (n = 513)	Obese Group (n = 100)	Non-Obese Group (n = 413)	*p* Value
Surgical approach				
Open, n (%)	142 (27.7%)	32 (32%)	110 (26.6%)	0.28
Laparoscopic, n (%)	268 (52.5%)	50 (50%)	218 (52.8%)	0.62
Robotic, n (%)	103 (20.1%)	18 (18%)	85 (20.6%)	0.56
Conversion, n (%)	70 (13.6%)	22 (22%)	48 (11.6%)	**0.007**
Skin incision				
Midline, n (%)	206 (40.2%)	53 (53%)	153 (37%)	**0.003**
Pfannenstiel, n (%)	231 (45%)	32 (32%)	199 (48.2%)	**0.004**
Other, n (%)	76 (14.8%)	15 (15%)	61 (14.8%)	0.95
Surgery type				
Anterior resection, n (%)	171 (33.3%)	31 (31%)	140 (33.9%)	0.58
Low anterior resection, n (%)	308 (60%)	60 (60%)	248 (60%)	0.99
Abdominoperineal resection, n (%)	32 (6.2%)	9 (9%)	23 (5.6%)	0.2
Additional procedure, n (%)	81 (15.8%)	19 (19%)	62 (15%)	0.33
Anastomosis, n (%)	17 (3.3%)	3 (3%)	14 (3.4%)	0.99
BSO, n (%)	40 (7.8%)	12 (12%)	28 (6.8%)	0.08
Hysterectomy, n (%)	10 (1.9%)	3 (3%)	7 (1.7%)	0.42
Hernia repair, n (%)	6 (1.2%)	1 (1%)	5 (1.2%)	0.99
Cholecystectomy, n (%)	3 (0.6%)	1 (1%)	2 (0.5%)	0.48
Intra-op complications, n (%)	80 (15%)	15 (15%)	65 (15.7%)	0.86
Bleeding/transfusion, n (%)	25 (4.9%)	5 (5%)	20 (4.8%)	0.99
Ureter/Urethra injury, n (%)	4 (0.8%)	1 (1%)	3 (0.7%)	0.58
Enterotomy, n (%)	22 (4.3%)	4 (4%)	18 (4.4%)	0.99
Splenic injury, n (%)	7 (1.7%)	0	7 (1.7%)	0.36
Vaginal injury, n (%)	8 (1.6%)	1 (1%)	7 (1.7%)	0.99
Anastomotic disruption, n (%)	8 (1.6%)	2 (2%)	6 (1.5%)	0.66
Stoma formation, n (%)	318 (61.9%)	65 (65%)	253 (61.3%)	0.49
Stoma reversal, n (%)	221 (69.5%)	45 (69.2%)	176 (69.6%)	0.96
Surgery duration (minutes) median (range)	270 (86–960)	300 (100–960)	260 (86–600)	**0.003**
LOS (days), median (range)	8 (3–98)	8 (5–98)	8 (3–86)	0.14
Postoperative complication, n (%)	339 (66.1%)	73 (73%)	266 (64.4%)	0.10
SSI, n (%)	78 (15.2%)	23 (23%)	55 (13.3%)	**0.02**
Abscess, n (%)	36 (7%)	5 (5%)	31 (7.5%)	0.38
Ileus/SBO, n (%)	111 (21.6%)	27 (27%)	84 (20.3%)	0.15
Anastomotic leak, n (%)	44 (8.6%)	7 (7%)	37 (9%)	0.53
Bleeding/transfusion, n (%)	71 (13.8%)	7 (7%)	64 (15.5%)	**0.03**
Pneumonia, n (%)	12 (2.3%)	5 (5%)	7 (1.7%)	0.06
UTI, n (%)	32 (6.2%)	8 (8%)	24 (5.8%)	0.42
DVT, n (%)	3 (0.6%)	0	3 (0.7%)	0.99
ACS/Arrythmia, n (%)	19 (3.7%)	5 (5%)	14 (3.4%)	0.39
Wound dehiscence, n (%)	10 (1.9%)	4 (4%)	6 (1.5%)	0.11
Electrolyte/ARF, n (%)	182 (35.5%)	40 (40%)	142 (34.4%)	0.29
Urinary retention, n (%)	38 (7.4%)	9 (9%)	29 (7%)	0.49
Clavien Dindo score, median (range)	1 (0–5)	1 (0–5)	1 (0–5)	0.25
Severe complications, n (%)	83 (16.2%)	16 (16%)	67 (16.2%)	0.96
30-day readmission, n (%)	91 (17.7%)	19 (19%)	72 (17.4%)	0.71
30-day mortality, n (%)	6 (1.2%)	1 (1%)	5 (1.2%)	0.99

Bold marks significance < 0.05. BSO—bilateral salpingo-oophorectomy, LOS—length of hospital stay, SSI—surgical site infection, SBO—small bowel obstruction, UTI—urinary tract infection, DVT—deep vein thrombosis, ACS—acute coronary syndrome, ARF—acute renal failure.

**Table 4:** Regression analysis for associations with severe complications.

	Univariate			Multivariate		
Variable	OR	95% CI	*p*	AOR	95% CI	*p*
Age	1.01	0.99–1.03	0.27	1.01	0.97–1.03	0.96
Female gender	0.59	0.36–0.96	**0.034**	0.67	0.34–1.33	0.25
BMI	1.03	0.98–1.08	0.27	1.04	0.98–1.11	0.19
ASA score	1.45	0.96–2.19	0.08	1.20	0.73–1.98	0.47
Distance from AV	0.92	0.87–0.97	**0.002**	0.92	0.84–1.01	0.08
Open surgical approach	2.18	1.34–3.54	**0.002**	2.57	1.39–4.73	**0.002**
Preop CEA	1.001	0.99–1.01	0.94	0.99	0.98–1.01	0.68
Preop Albumin	0.46	0.26–0.82	**0.008**	0.45	0.21–0.94	**0.03**
Preop Creatinine	2.35	0.99–5.58	0.054	2.10	0.63–7.02	0.23
Pathological stage	1.03	0.83–1.27	0.78	1.10	0.85–1.43	0.48
Preop Radiotherapy	1.43	0.89–2.29	0.13	1.34	0.37–4.87	0.65
Preop Chemotherapy	1.22	0.76–1.95	0.41	0.46	0.13–1.59	0.22

Bold marks significance < 0.05. AOR—adjusted odds ratio, BMI—body mass index, ASA—American Society of Anesthesiologists, AV—anal verge, CEA—carcinoembryonic antigen.

**Table 5:** Pathological results.

Variable	All Cohort (n = 513)	Obese Group (n = 100)	Non-Obese Group (n = 413)	*p* Value
Pathological complete response, n (%)	54 (10.5%)	11 (11%)	43 (10.4%)	0.86
Pathological tumor size (cm), mean (range)	3.06 (0–11)	3.45 (0.2–10.2)	2.96 (0–11)	0.075
Tumor differentiation				
Well-differentiated, n (%)	134 (26.1%)	28 (28%)	106 (25.7%)	0.63
Moderately differentiated, n (%)	215 (41.9%)	46 (46%)	169 (40.9%)	0.36
Poorly differentiated, n (%)	19 (3.7%)	2 (2%)	17 (4.1%)	0.31
Pathological staging				
0, n (%)	54 (10.5%)	11 (11%)	43 (10.4%)	0.86
I, n (%)	173 (33.7%)	34 (34%)	139 (33.7%)	0.95
II, n (%)	122 (23.8%)	28 (28%)	94 (22.8%)	0.27
III, n (%)	154 (30%)	24 (24%)	130 (31.5%)	0.14
IV, n (%)	5 (1%)	2 (2%)	3 (0.7%)	0.24
Pathological T staging				
0, n (%)	55 (10.7%)	11 (11%)	44 (10.7%)	0.92
1, n (%)	62 (12%)	12 (12%)	50 (12.1%)	0.98
2, n (%)	139 (27.1%)	26 (26%)	113 (27.4%)	0.78
3, n (%)	240 (46.8%)	50 (50%)	190 (46%)	0.47
4, n (%)	10 (1.9%)	0	10 (2.4%)	0.22
Number of nodes examined, median (range)	15 (0–113)	16 (2–62)	14 (0–113)	0.38
Pathological N staging				
0, n (%)	349 (68%)	74 (74%)	275 (66.6%)	0.15
1, n (%)	126 (24.6%)	20 (20%)	106 (25.7%)	0.24
2, n (%)	31 (6%)	5 (5%)	26 (6.3%)	0.63
LVI, n (%)	38 (7.4%)	4 (4%)	34 (8.2%)	0.15
Perineural invasion, n (%)	39 (7.6%)	2 (2%)	37 (9%)	**0.018**
Distal margin involved, n (%)	6 (1.2%)	3 (3%)	3 (0.7%)	0.09
Distance from distal margin (cm), mean (range)	2.76 (0.14–9)	2.43 (0.25–6)	2.76 (0.14–9)	0.33
Radial margin involved, n (%)	5 (1%)	0	5 (1.2%)	0.59
Signet ring, n (%)	11 (2.1%)	2 (2%)	9 (2.2%)	0.99
Mucin producing, n (%)	87 (17%)	17 (17%)	70 (16.9%)	0.99

Bold marks significance <0.05.

**Table 6:** Cox regression analysis for associations with disease recurrence.

	Univariate			Multivariate		
Variable	HR	95% CI	*p*	HR	95% CI	*p*
Age	1.005	0.99–1.02	0.49	1.01	0.99–1.03	0.19
Female Gender	0.97	0.69–1.37	0.87	0.86	0.54–1.38	0.54
ASA score	1.19	0.88–1.63	0.25	1.09	0.95–1.05	0.63
BMI	0.98	0.95–1.02	0.42	0.99	0.90–1.04	0.78
Distance from AV	0.95	0.92–0.99	**0.023**	0.97	0.9–1.04	0.36
Pathological stage	1.33	1.22–1.46	**<0.001**	1.33	1.17–1.52	**<0.001**
Preop CEA	1.01	1.005–1.02	**<0.001**	1.01	0.99–1.02	0.32
Preop Albumin	0.46	0.30–0.70	**<0.001**	0.66	0.39–1.09	0.11
Preop Creatinine	1.36	0.68–2.73	0.38	1.09	0.46–2.59	0.85
Open surgical approach	1.24	0.85–1.8	0.26	0.98	0.60–1.60	0.95
Severe complication	1.2	0.76–1.9	0.43	0.72	0.38–1.36	0.31
Preop Radiotherapy	1.08	0.77–1.52	0.66	0.46	0.09–2.41	0.36
Preop Chemotherapy	1.18	0.84–1.65	0.35	2.2	0.42–11.0	0.36

Bold marks significance <0.05. ASA—American Society of Anesthesiologists, BMI—body mass index, AV—anal verge, CEA—carcinoembryonic antigen.

## Data Availability

The raw data supporting the conclusions of this article will be made available by the authors on request.

## Supplementary Materials

The following supporting information can be downloaded at: https://www.mdpi.com/article/10.3390/jcm15072693/s1, Figure S1: CONSORT flow diagram depicting the selection process, inclusion, and exclusion of patients undergoing radical rectal resection.
