# Supplementary figures and images for: The Impact of Obesity on Operative Outcomes and Long-Term Oncological Outcomes Following Rectal Cancer Surgery: A Retrospective Single-Center Study
Source: J Clin Med. 2026 Apr 2;15(7):2693. doi: 10.3390/jcm15072693 (PMC13074016; doi:10.3390/jcm15072693)

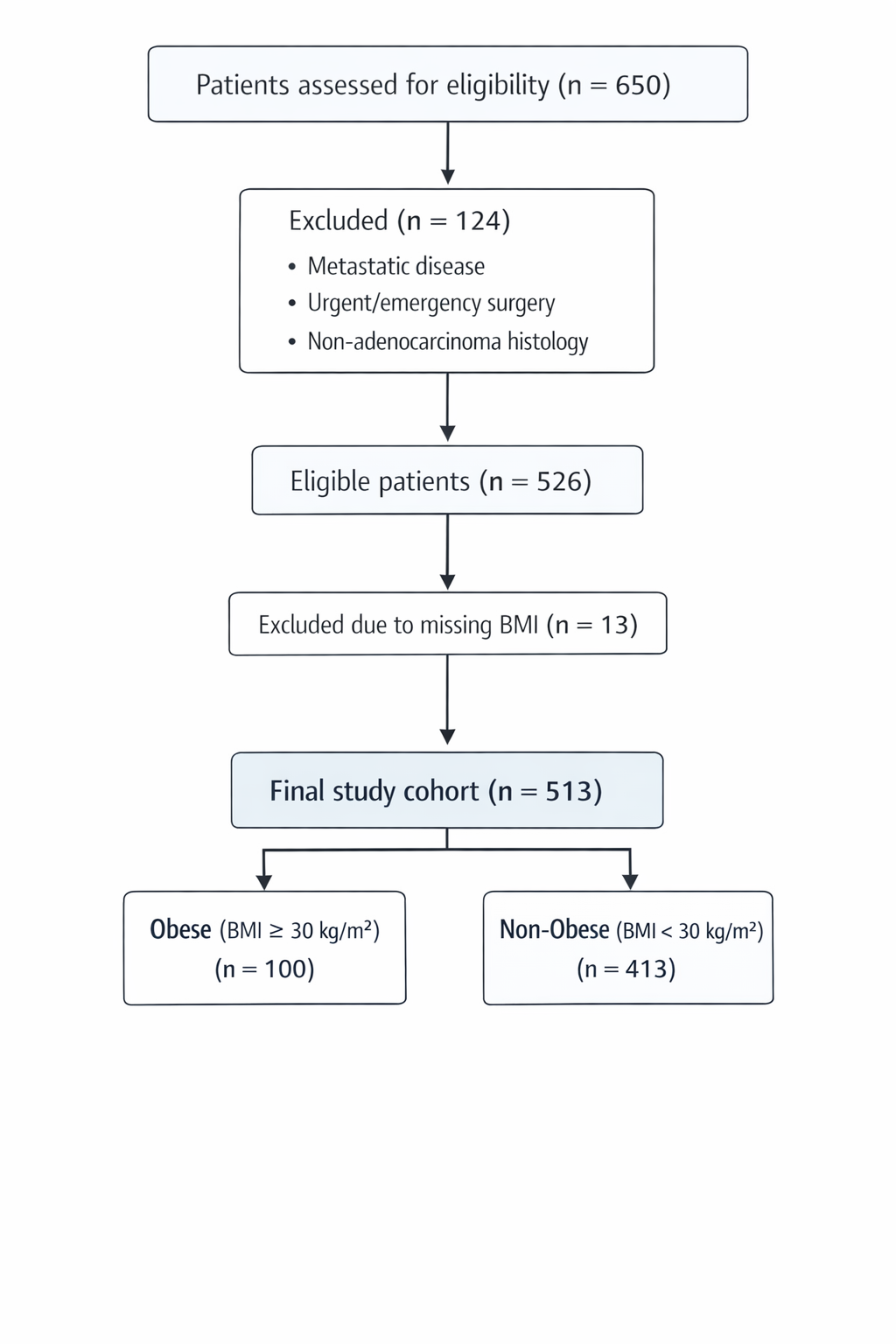

Supplement: Supplementary file 1 [file jcm-15-02693-s001.zip › jcm-4183806-supplementary.png]
